# Critical Overview on Regenerative Medicine: New Insights into the Role of Stem Cells and Innovative Biomaterials

**DOI:** 10.3390/ijms24097936

**Published:** 2023-04-27

**Authors:** Marco Tatullo, Barbara Zavan, Adriano Piattelli

**Affiliations:** 1 Department of Translational Biomedicine and Neuroscience (DiBraiN), University of Bari Aldo Moro, 70124 Bari, Italy; 2 University of Dundee, Dundee DD1 4HR, UK; 3 MIRROR—Medical Institute for Regeneration and Repairing and Organ Replacement, Interdepartmental Center, School of Medicine, University of Bari Aldo Moro, 70124 Bari, Italy; 4 Translational Medicine Department, University of Ferrara, 44121 Ferrara, Italy; barbara.zavan@unife.it; 5 School of Dentistry, Saint Camillus International University of Health and Medical Sciences, Via di Sant’Alessandro 8, 00131 Rome, Italy; apiattelli51@gmail.com

Regenerative medicine represents a novel and intriguing field of medicine. A growing number of regenerative procedures are based on innovative biomaterials and newly discovered stem cells, highlighting the degree to which this branch of medicine is continuously improving and evolving. Stem cells are unique cells that have the potential to differentiate into various types of specialized cells and they can be obtained from different sources such as embryos, adult tissues, and induced pluripotent stem cells (iPSCs). Conversely, stem cells require scaffolds to improve their biological effects; innovative biomaterials such as hydrogels and nanoparticles can be used to support stem cell growth and differentiation, and they can also be engineered to release growth factors or drugs to promote tissue regeneration in several human tissues. Both the development of biomimetic scaffolds that mimic the structural and functional characteristics of native tissue and the potential of stem cells to enhance tissue regeneration have been successfully investigated for potential applications in osteochondral regeneration, bone tissue engineering [1,2], and for myocardial tissue engineering. Currently, the extant scientific literature suggests that the development of biomimetic scaffolds [3] for tissue engineering and the use of stem cells to enhance tissue regeneration hold extraordinary promise for the treatment of a wide range of conditions.

In recent years, several researchers have discussed the potential of using mesenchymal stem cells (MSCs) to regenerate different tissues, including bone, cartilage, and other human tissues; moreover, novel materials have been investigated and coupled with MSCs in order to improve the utilization of the biomimetic approach in human prosthetics and in biomedical devices [4]. The challenges of using MSCs in bone and dental applications have been addressed by using various types of stem cells, such as dental pulp, periodontal ligament, and human–periapical odontogenic cysts [5]. The association of MSCs and innovative biomaterials [6,7] is the modern strategy to make regenerative medicine applicable to organs and tissues which have either been damaged or undergone degenerative processes. Nevertheless, stem cell transplantation has also been investigated in cases of severe diseases, such as cancers [8].

Thus, the recent period has seen the field of regenerative medicine become oriented around the roles of stem cells and innovative biomaterials. Owing to the extensive research conducted into these subjects, many significant advances have been made to date. In this landscape, this Special Issue of *IJMS*, entitled “Regenerative Medicine: Role of Stem Cells and Innovative Biomaterials”, highlights the most promising applications of tissue engineering in the future diagnostic and therapeutic procedures designed for application to medical science.

One common theme among the conclusions drawn in the 10 articles published in this Special Issue concerns the critical role of molecular and cellular processes in maintaining overall health and preventing various diseases. For instance, Chisa Katou-Ichikawa et al. [9] highlight the role of A3-labeled somatic stem cells in the process of cutaneous wound healing, with a higher density of A3-labeled cells being observed in the wound bed during the healing process.

Aesthetics in healing processes is an important topic, one which is also considered in the article by Conti et al. [10]. The authors present findings that suggest an alteration in the extracellular matrix of skin affected by cellulite, as well as changes in the abundance of proteins involved in adipose tissue metabolism and inflammation. Conversely, Dai Prè et al. [11] indicate a method for isolating healthy adipose stem cells through non-enzymatic extraction, obtaining evidence that this is a simple and effective method with various applications in regenerative medicine.

On the other hand, the research focuses on clinical needs, such as the cardiovascular diseases. Fang et al. [12] find that the iPSCs derived from hAFSCs have a low expression of MHC molecules, suggesting that they may have immune privilege and may not elicit an immune response when transplanted into a recipient. Overall, the study provides evidence that could be beneficial for transplantation purposes.

Undoubtedly, nerve repair can be considered an important clinical and multifactorial issue; Bonnet et al. [13] report interesting insights into the effects of immediate or delayed transplantation of a vein conduit filled with nasal olfactory stem cells on rats with a peroneal nerve injury. The results seem to suggest that nasal olfactory stem cells could be a promising treatment option for nerve injuries in humans.

The most investigated topic encompasses the regenerative and reparative procedures applied to bone tissue. Kim et al. [14] report the use of bone marrow aspirate concentrate (BMAC) to treat osteoarthritis, with promising results, including reduced pain and improved joint function in patients with osteoarthritis. Of course, there are some bone diseases that need bone implantation to obtain a restoration of anatomy and function; Tumedei et al. [15] provide a systematic review and meta-analysis of the use of synthetic blocks for bone regeneration, suggesting that synthetic blocks could be a valuable tool for clinicians and researchers working in the field of bone regeneration. On the other side, Luca et al. [16] focus their interest on the osteogenic potential of bovine bone graft combined with laser photobiomodulation (LPBM) in rats; interestingly, the study suggests that this combination could be a promising treatment option for bone defects in humans, particularly for patients with osteoporosis or bone fractures. Lastly, where self-repairing procedures are not able to improve the clinical issues, the use of novel scaffolds is recommended. Ehlert et al. [17] first report preliminary in vitro testing of a titania nanofiber scaffold for tissue engineering applications, suggesting that the titania nanofiber scaffold has potential for use in tissue engineering applications due to its enhanced biointegration activity.

In summary, these articles highlight the critical importance of stem cells and their biological processes in maintaining overall health and preventing various diseases. In conclusion, it is worth highlighting the research by Rehakova et al. [18]: the authors emphasize the need for a standardized approach to characterizing clinical-grade human pluripotent stem cells (hPSCs) for use in cell therapy; importantly, a thorough characterization strategy is crucial for developing safe and effective hPSC-based therapies, and collaboration among scientists, clinicians, and regulatory bodies is essential to achieve this goal.

Overall, the described articles contribute significantly to the field of regenerative medicine by highlighting the potential of stem cells and innovative biomaterials for use in tissue regeneration. They also address some of the key challenges associated with the translation of these therapies to the clinic, such as the need to develop a better understanding of stem cell behavior and the need for more efficient biomaterial-based delivery systems. The findings of these studies have the potential to lead to the development of new and more effective regenerative therapies that can improve the lives of patients suffering from various diseases and injuries.
